# Supplementary material for: Barriers and Facilitators to Healthy Eating for Shift-Work-Registered Nurses in Hong Kong Public Hospitals: An Exploratory Multi-Method Study
Source: Nutrients. 2025 Mar 27;17(7):1162. doi: 10.3390/nu17071162 (PMC11990088; doi:10.3390/nu17071162)
Supplement: Supplementary file 1 [file nutrients-17-01162-s001.zip › nutrients-3515649-supplementary.pdf]

nutrients

MDPI

Study ID: ________________________ Date of Interview: __________________________ ☐FFQ1 ☐FFQ2

A. Your taste preference over the past **ONE month**: ☐Light(2) ☐Normal(4) ☐Salty(6)

B. Please tell us the **average consumption** if you have eaten the following foods over the past **ONE month.**
(Please √ the frequencies and fill in the portions according to the food photo booklet):

(Note: 1 bowl=300ml；1 cup=240ml；1 Chinese Tablespoon=20ml；1 Teaspoon=5ml)

Type of Food	Never	1 time per month	1-3 times per month	1 time per week	2 times per week	3-4 times per week	5-6 times per week	1 time per day	2 or more times per Day	Average portion each time	Reference portion	Code	Amount per day	
1. Refined grains, cooked (regular)										bowl	1 bowl=200g			8
2. Refined grains, cooked (soft or thin)										bowl	1 bowl=200g			8
3. Whole grain, cooked										bowl	1 bowl=200g			8
4. Refined bread										slice/piece (circle the unit)	1 slice=50g 1 piece=70g			8
5. Whole grain bread										slice/piece (circle the unit)	1 slice=40g 1 piece=70g			8
6. Refined cereals, dry										bowl	1 bowl=40g(dry)			8
7. Whole grain cereals, dry										bowl	1 bowl=40g(dry)			8
8. Fresh fruits										cup/piece	1cup/piece=150g			8
9. Dried fruits										C Tbsp	1 C Tbsp=15g			8
10. Tomatoes										serving	1 serving=100g			8
11. Prebiotic vegetables										C Tbsp	1 C Tbsp=10g			8
12. Root and tuber vegetables										serving	1 serving=100g			8
13. Other vegetables (leaf/squash/fruit)										bowl	1 bowl=150g			8

Figure S1 *Sample page of the Food Frequency Questionnaire (FFQ)*

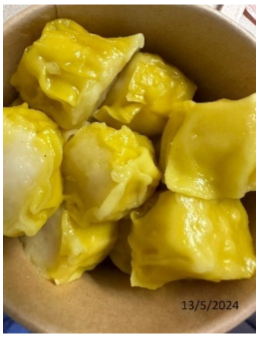

Figure S2a

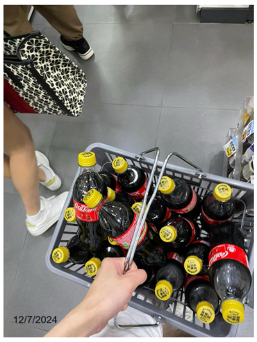

Figure S2b

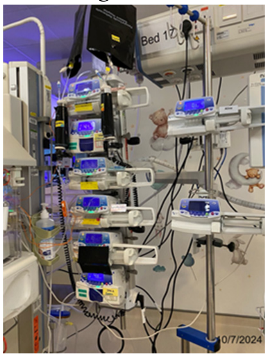

Figure S2c

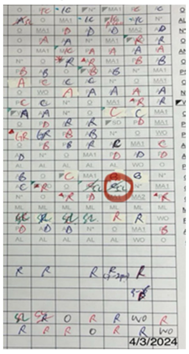

Figure S2d

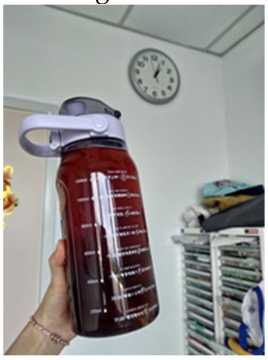

Figure S2e

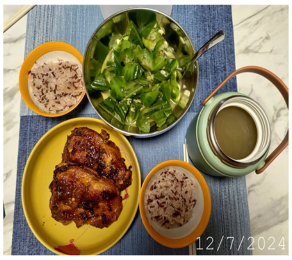

Figure S2f

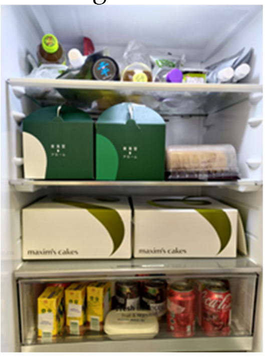

Figure S2g

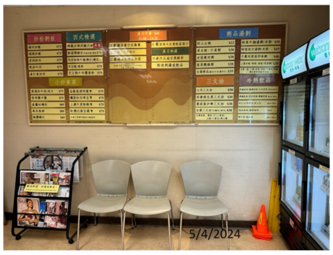

Figure S2h

Figure S2 *Photo Album*
